# Genetic variants in the calcium signaling pathway participate in the pathogenesis of colorectal cancer through the tumor microenvironment

**DOI:** 10.3389/fonc.2023.992326

**Published:** 2023-02-07

**Authors:** Jing-Yu Wu, Yu Shao, Chang-Zhi Huang, Zhen-Ling Wang, Hong-Qiang Zhang, Zan Fu

**Affiliations:** The General Surgery Laboratory, The First Affiliated Hospital of Nanjing Medical University, Nanjing, China

**Keywords:** the calcium signaling pathway, PDE1C, colorectal cancer, genetic variants, immunotherapy

## Abstract

**Background:**

Cancer risk is influenced by calcium signaling in intracellular and intercellular signaling pathways. However, the relationship between the calcium signaling pathway and colorectal cancer risk remains unknown. We aim to evaluate the role of genetic variants in calcium signaling pathway genes in colorectal cancer risk through the tumor microenvironment.

**Methods:**

An analysis of genetic variants in the calcium signaling pathway was conducted using a case-control study that included 1150 colorectal cancer patients and 1342 non-cancer patients. Using the regression model, we assessed whether single-nucleotide polymorphisms (SNPs) increase the risk of colorectal cancer. We also performed a dual luciferase reporter gene assay using HCT116 cell lines and DLD1 cell lines to demonstrate the regulatory relationship between SNP and candidate risk gene. We evaluated the expression of candidate risk gene in different populations. In addition, we also evaluated candidate risk gene and 22 immune cells correlation studies.

**Results:**

There was a significant association between the *PDE1C* rs12538364 T allele and colorectal cancer risk [odds ratio (OR) = 1.57, 95% confidence interval (CI) = 1.30 – 1.90, *P* = 3.07 × 10^–6^, *P*_FDR_ = 0.004]. Mutation of intron region rs1538364 C to T locus reduces promoter activity of *PDE1C* in DLD1 and HCT116 cell lines (*P* < 0.05). We identified that *PDE1C* is significantly down-regulated in colorectal cancer, closely associated with 22 immune cells. Finally, we found that *PDE1C* could be the biomarker for individual immunotherapy of colorectal cancer.

**Conclusion:**

According to our findings, PDE1C may be a key factor contributing to colorectal cancer, thus improving individual immunotherapy for the disease. The potential mechanism by which polymorphisms in the calcium signaling pathway genes may participate in the pathogenesis of colorectal cancer through the tumor microenvironment.

## Introduction

Worldwide, colorectal cancer remains a major public health threat. In 2021, around 149,500 persons will be diagnosed with colorectal cancer, accounting for approximately 8% of all new cases (1). The current standard of treatment for individuals with colorectal cancer is a combination of surgery, radiation, and chemotherapy (2), which are insufficient to prevent colorectal cancer. Immunotherapy for colorectal cancer has advanced (3), however, resistance to immunotherapy nevertheless occurs on occasion (4). Numerous variables contribute to the emergence and progression of colorectal cancer during its multiple stages (5). However, these characteristics do not fully explain the development of colorectal cancer. At the moment, a large majority of published studies have shown an association between single-nucleotide polymorphisms (SNPs) and the development of colorectal cancer (6).

A rising body of data suggests that calcium signaling is intimately associated with cancer (7, 8). Somatic processes such as cell development and proliferation and death are all thought to be influenced by calcium (9, 10). Calcium signaling has been related to a variety of cellular activities since Hanahan and Weinberg suggested six cancer hallmarks in 2000 (11). When taken regularly, calcium supplements may help lower the risk of colorectal cancer (12). It has been suggested that cancer may be a reverse abnormality of several normal processes involving calcium. The process is triggered or regulated by calcium influx across distinct cellular compartments (13). Recent studies have shown that the calcium signaling pathways components are rearranged or unregulated in cancer. Colorectal cancer has been connected to a transient receptor potential channel called *TRPM8 (* 14).

NK and T lymphocytes, tumor-associated macrophages, dendritic cells (DCs), and cancer-associated fibroblasts are all found in the tumor microenvironment (CAFs). The tumor microenvironment plays an important role in cancer development. The calcium signaling route to cancer cells may be mediated by the tumor microenvironment (e.g. immune cells, cancer-associated fibroblast produced substances) (15). However, the majority of the procedure is unclear at this stage.

The purpose of this research was to determine if genetic variants in calcium signaling pathway genes were associated with an increased risk of colorectal cancer. Based on the Chinese population, we assessed the influence of tagged SNPs on colorectal cancer risk. We also explored the tumor microenvironment to see whether there was an association between genetic variants and colorectal cancer risk.

## Materials and methods

### Study population

1,150 colorectal cancer patients were included in this research, whereas 1,342 cancer-free controls were included as well. Beginning in September 2010, all of the patients were treated in the First Affiliated Hospital of Nanjing Medical University and the Affiliated Nanjing Hospital of Nanjing Medical University, which are both affiliated with Nanjing Medical University. Patients having a history of inflammatory bowel illness, colorectal neoplasia, or any other malignancy were excluded from the study. The control participants were chosen at random from a pool of more than 25,000 patients who had had a regular physical examination, and their age (less than 5 years) and sex distributions were matched to those of the patients (16). The research population’s demographics and other features have already been reported in detail. For the research and publishing of their samples and personal information, all study participants signed a written informed permission form. The Nanjing Medical University Ethics Committee authorized our study.

### Selection of genes and single−nucleotide polymorphisms (SNPs)

Candidate genes for the calcium signaling pathway were chosen by the Kyoto Encyclopedia of Genes and Genomes (KEGG) (http://www.kegg.jp ) (Kobe, Japan). According to the Cancer Genome Atlas (TCGA) database (http://cancergenome.nih.gov/ ), genes with statistically significant differential expression in colorectal cancer were maintained for future investigation. Data from the Han Chinese in Beijing (CHB) and Japanese in Tokyo (JPT) populations, as well as data from the 1000 Genomes Project, were used to identify SNPs in these genes situated at positions 2 kb upstream to 2 kb downstream of the coding region. Given that the majority of disease-associated SNPs are common mutations, we selected high-quality SNP sites based on the following criteria: SNPs located on autosomal chromosomes, a minor allele frequency (MAF) ≥ 0.05, a Hardy–Weinberg equilibrium (HWE) *P*-value ≥ 0.05, and a call rate > 95%. Based on the sample size in this investigation, we established the MAF threshold at 0.05 in the aforementioned criterion. Using the PLINK 1.90 software, we carried out a linkage disequilibrium (LD) analysis to identify the tagged SNPs (*r* ^2^ ≥ 0.8) for further study.

### Functional analysis

We utilized ENCODE (https://www.encodeproject.org/ ) to identify histone modifications associated with risk SNPs and then visualized the results using the UCSC genome browser (http://genome.ucsc.edu/ ). RNAfold (https://rna.tbi.univie.ac.at/cgi-bin/RNAWebSuite/RNAfold.cgi/ ) was used to predict RNA secondary structure and determine the protective and risk allele’s minimal free energy (MFE). A protein-protein interaction network was created using the Search Tool for the Retrieval of Interacting Genes (STRING, https://string-db.org/ ). The summary score was supplied by RegulomeDB (https://www.regulomedb.org/ ).

### Cell culture

Two different types of intestinal cancer cells including HCT116 and DLD1 were purchased from the Chinese Academy of Sciences (Shanghai Cell Institute). DLD1 cell was grown in Roswell Park Memorial Institute-1640 (Procell, PM150110). HCT116 was grown in DMEM/F12 (Procell, PM150312). Media were supplemented with 10% fetal bovine serum (Biological Industries, C04001-500) and 100 μg/mL penicillin-streptomycin solution (Procell, PB180120). Cells were grown at 37 °C in a humid incubator with 5% CO2.

### Luciferase activity

HCT116 and DLD1 cells were inoculated into six-well plates. When cultured to 70% density, Lipofectamine 3000 (Lifetech, USA) and reporter plasmids were added for transfection. After 24 hours, the cells were collected by trypsin digestion (beyotime,China) and the fluorescence intensity was measured using the Dual-Luciferase Reporter Assay System (Promega, USA).

### Gene expression analysis and expression quantitative trait loci (eQTL) analysis

We performed the eQTL analysis to verify the relationship between genetic variants and mRNA expression levels. 625 colorectal cancer tissue samples and 50 normal colorectal tissue samples were retrieved from the TCGA database (The Cancer Genome Atlas Program - National Cancer Institute). The Gene Expression Omnibus (GEO2) database (https://www.ncbi.nlm.nih.gov/) was used to obtain the Affymetrix microarray-based gene expression matrix, which included 144 colorectal cancer patients in accession GSE21510 (17), 32 colorectal cancer patients in accession GSE8671 (18), and 69 colorectal cancer patients in accession GSE9348 (19). Paired and unpaired Student’s *t*-tests were used to examine the differential expressions of potential genes (log_2_(FPKM+1) transformed). To show the change in the mRNA expression level of the candidate gene, data from the Gene Expression Omnibus (GEO, https://www.ncbi.nlm.nih.gov/gds/) database (GSE9348, GSE8671, and GSE21510) were utilized.

### Calculation of the immune cell infiltration

We utilized CIBERSORT (20) to assess the relative number of 22 types of immune cells. CIBERSORT is a deconvolution technique that predicts the cell composition of complicated tissues using supporting vector regression based on the gene expression profiles of a large number of tumor samples using a set of reference gene expression values as the minimal representation of each cell type. Newman’s (http://cibersort.stanford.edu/publication) provides a full explanation of the 22 immune cell types (20). The R package “corrplot” was used to analyze and visualize the landscape of the relative fraction of the 22 immune cells.

### Estimation of stromal and immune cells in malignant tumors using expression data (ESTIMATE)

The immune purity was calculated using the R package “estimate” using the mRNA expression matrix of colorectal cancer samples from TCGA samples. After each sample was generated using the ssGSEA(single sample gene set enrichment analysis) technique, the stromal and immune scores, as well as the estimation score, were calculated independently for each subgroup. The estimated score was calculated by combining the stromal and immune scores for a specific sample, and it was used to determine tumor purity.

### The investigation of the TME and immune checkpoints

TIMER2.0 (http://timer.cistrome.org/), which contained the TIMER, CIBERSORT, XCELL, QUANTISEQ, MCPcounter, EPIC, and CIBERSORT, is used to quantify immune infiltration statuses among colorectal cancer patients from the TCGA. Alternatively, we utilize the same website to estimate infiltration profiles for all TCGA tumors. To investigate the differences in immune infiltrating cell concentration, the Wilcoxon signed-rank test, limma, scales, ggplot2, and ggtext R package were used. The bubble chart was used to display the findings (21). We also used the “ggpubr” R package to compare TME scores and immune checkpoint activation between low and high-mRNA levels of *PDE1C*.

### Statistical analysis

The difference in demographic distribution between 1,150 cases and 1,342 controls was determined using Student’s *t* and Chi-square tests. We used logistic regression models to determine the odds ratios (OR) and 95% confidence interval (CI) associated with genetic variations and colorectal cancer risk. The control group’s Hardy-Weinberg equilibrium was evaluated using the Chi-square test. The false discovery rate (FDR) was used for *P*-value modification to lower the false-positive rate after multiple comparisons. This helped to minimize the false-positive rate after multiple comparisons. We used demographic and clinicopathological variables to stratify. The Mann-Whitney Wilcoxon test was performed to evaluate the percentages of immune cells in the high and low expression groups of patients. The Spearman correlation coefficient was utilized to examine the relationship between the anticipated transcription factor motif and the target genes. The association between the stromal score, the immunological score, the estimated score, and the target genes was investigated using the Spearman technique. The Mann-Whitney Wilcoxon test was employed to compare immune cell fractions between patients with high and low target gene expression RNA-seq. All analyses used R 4.0.2 and PLINK 1.90 while *P* < 0.05 were deemed statistically significant.

## Results

### Association between SNPs in the key genes and CRC

**Figure 1:**
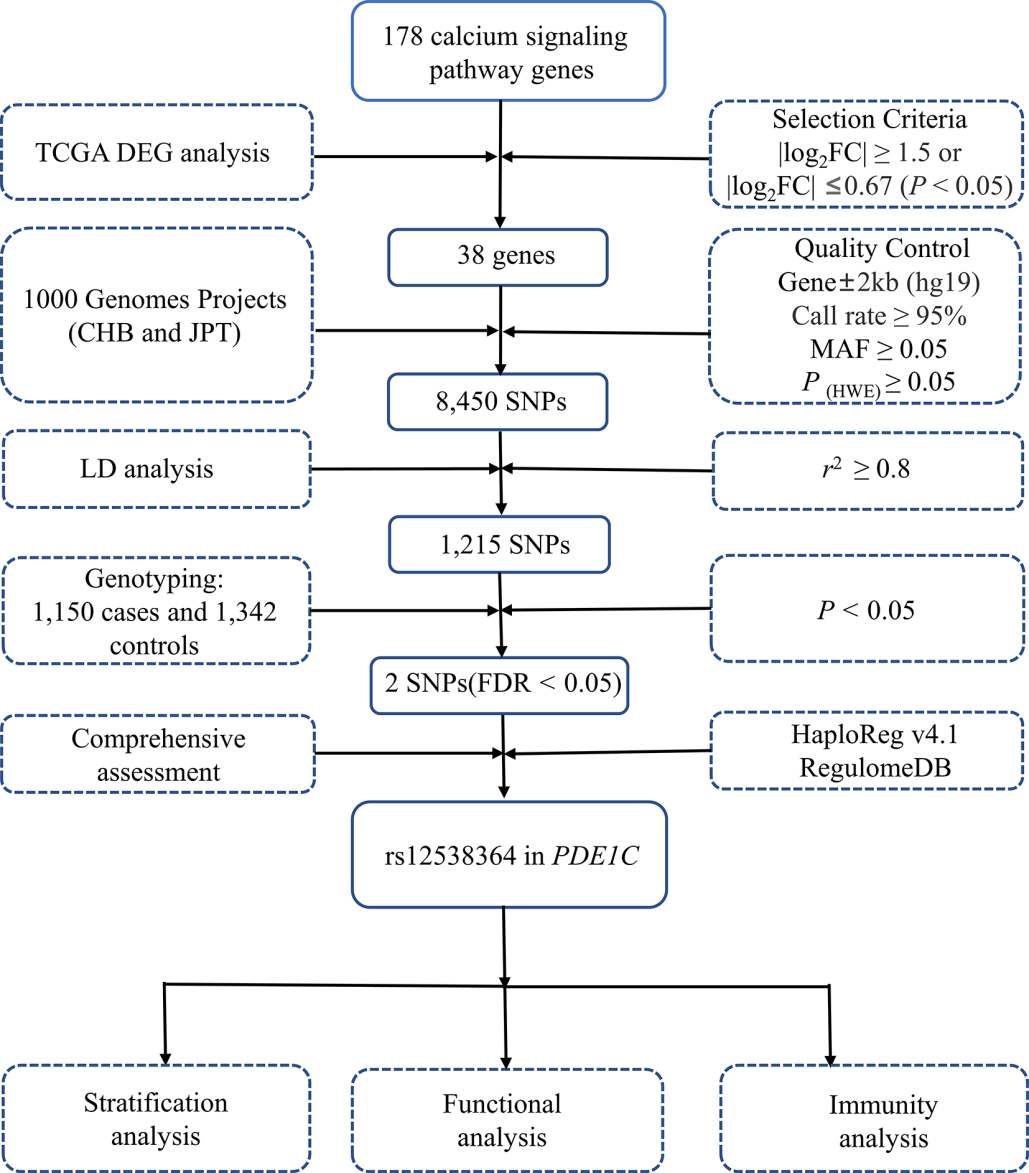
Flow diagram of SNP selection. The workflow of the analysis includes the screening criteria and the methods. MAF, minor allele frequency; HWE, Hardy-Weinberg equilibrium; LD, linkage disequilibrium.

To begin with, 38 key genes were found to be significant in both the TCGA and GEO datasets (*P* < 0.05, **Supplementary Table 1**). As shown in the **Supplementary Figure 1**, 38 key genes are statistically relevant (*P* < 0.05). After the quality screening, 8450 SNPs from the 1000 Genomes Project were chosen. Independent 1215 SNPs were retained after LD analysis (**Figure 1**). Only rs12538364 and rs12913815 remained after susceptibility analysis in the additive model and multiple **c**orrections (*P*_FDR_ < 0.05, **Supplementary Table 2**). After function annotation (*P* < 0.05, **Supplementary Table 3**), only SNP rs12538364 remained significant in *PDE1C* in the Chinese population after function annotation (OR = 1.57, 95% CI = 1.30-1.90, *P* = 3.07×10^-6^, *P*_FDR_ = 0.004; **Supplementary Table 2**).

The rs12538364 T allele increased the risk of colorectal cancer in the codominant genetic model in the Chinese population after full adjustment for age and sex (OR = 1.62, 95% CI = 1.32-2.00, *P* = 4.45×10^-6^, **Table 1**). *PDE1C* rs12538364 CT/TT genotypes were linked with an elevated risk in the dominant genetic model in the Chinese population (OR = 1.63, 95% CI = 1.33-1.20, *P* = 2.19×10^-6^) as compared to the reference genotype. The effect of the rs12538364 T allele on colorectal cancer susceptibility in the Chinese population was not significant in the recessive genetic model.

**Table 1:** Association analysis between PDE1C rs12538364 and colorectal cancer risk in the Chinese population.

Genotype	Cases	Controls	OR (95%CI)^a^	*P* ^a^
CC	886	1,125	1.00	
CT	247	193	1.62 (1.32-2.00)	4.45 ×10^-6^
TT	13	9	1.83 (0.78-4.31)	1.63 ×10^-1^
Additive model			1.57 (1.30-1.90)	3.07 ×10^-6^
Dominant model			1.63 (1.33-2.00)	2.19 ×10^-6^
Recessive model			1.68 (0.71-3.94)	2.33 ×10^-1^

OR odds ratio, CI confidence interval

^a^Adjusted for age and sex in the logistic regression model

### Stratification analysis of rs12538364 with CRC risk

As shown in **Table 2** and **Supplementary Figure 2**, According to statistical analysis, unfavorable genotypes are significantly associated with colorectal cancer risk in age (OR = 1.45,95%CI = 1.12-1.97, *P* = 5.74 × 10^-3^ for age ≤ 60 and OR = 1.69,95%CI = 1.30-2.19, *P* = 8.16×10^-5^ for age > 60), sex (OR = 1.66,95%CI = 1.29-2.13, *P* = 8.48×10^-5^ for male and OR = 1.48,95%CI = 1.10-1.98, *P* = 9.45×10^-3^ for female), smoking status (OR = 1.69,95%CI = 1.33-2.51, *P* = 1.95×10^-5^ for non-smoker and OR = 1.36,95%CI = 0.99-1.86, *P* ^a^ = 5.95×10^-2^ for smoker) and drinking status (OR = 1.47,95%CI = 1.18-1.84, *P* = 7.71×10^-4^ for non-drinker and OR = 1.91,95%CI = 1.32-2.76, *P* = 6.60×10^-4^ for drinker). A similar tendency was found in subgroup analyses of clinicopathologic characteristics. Patients with poor tumor grade had a substantially higher risk than those with good or moderate tumor grade (OR = 1.62,95%CI = 1.14-2.31, *P* = 7.70×10^-3^, **Supplementary Table 4**). Moreover, a significant association was identified in colon (OR = 1.62,95%CI = 1.29-2.03, *P* = 3.64×10^-5^) and Duck Stage A and B (OR = 1.65,95%CI = 1.31-2.08, *P* = 2.83×10^-5^).

**Table 2:** Stratified analyses of demographic characteristics for the association between *PDE1C* rs12538364 and colorectal cancer risk.

Variables	Category	Cases			Controls			Adjusted OR (95%CI)^a^	*p^a^*	*p^b^*
		CC	CT	TT	CC	CT	TT			
Age	≤60	474 (79.7)	115 (19.3)	6 (1.0)	531 (85.8)	84 (13.5)	4 (0.6)	1.45 (1.12,1.97)	5.74 x 10^-3^	0.435
	> 60	412 (74.8)	132 (24.0)	7 (1.2)	594 (83.9)	109 (15.4)	5 (0.7)	1.69 (1.30,2. 19)	8.16 x 10^-5^	
Sex	Male	529 (77.3)	148 (21.6)	7 (1. 1)	668 (85.6)	106 (13.6)	6 (0.8)	1.66 (1.29,2.13)	8.48 x 10^-5^	0.560
	Female	357 (77.2)	99 (21.4)	6 (1.4)	457 (83.5)	87 (15.9)	3 (0.6)	1.48 (1.10,1.98)	9.45 x 10^-3^	
Smoking status	No	582 (77.8)	158 (21.1)	8 (1. 1)	769 (86. 1)	118 (13.2)	6 (0.7)	1.69 (1.33,2.51)	1.95 x 10^-5^	0.341
	Yes	304 (76.4)	89 (22.4)	5 (1. 1)	356 (82.0)	75 (17.3)	3 (0.7)	1.36 (0.99,1.86)	5.95 x 10^-2^	
Drinking status	No	620 (77.6)	172 (19.1)	7 (3.3)	818 (84.2)	145 (14.9)	8 (0.9)	1.47 (1.18,1.84)	7.71 x 10^-4^	0.233
	Yes	266 (76.7)	75 (21.6)	6 (1.7)	307 (86.2)	48 (13.5)	1 (0.3)	1.91 (1.32,2.76)	6.60 x 10^-4^	

OR, odds ratio; CI, confidence interval.

^a^Adjusted for age and sex in logistic regression model.

^b^ *P* value for the heterogeneity.

### Functional annotation

RNA secondary structure images of protective and risk allele are shown in **Supplementary Figure 3A**. The change in the minimum free energy (MFE) caused by rs12538364 C allele to rs12538364 T allele was predicted by RNAfold. ΔMFE was obtained by subtracting the wild-type MFE from the mutant MFE (ΔMFE = 3.0 kcal/moL). Additionally, the region surrounding rs12538364 in HCT-116 was functionally annotated. Multiple histone (H2AFZ, H3k27ac, H3k27me3, H3k36me3, H3K4me1, and H3K4me2) modification peaks were obtained from ENCODE and visualized by UCSC genome browser (**Supplementary Figure 3B**).

### The *PDE1C* mRNA expression levels analysis

**Figure 2:**
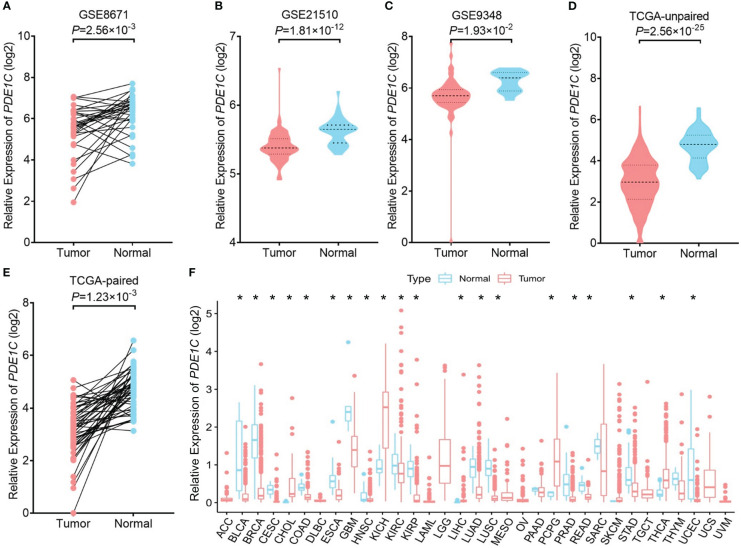
mRNA expression levels of PDE1C in cancer and para cancer tissue. The expression of PDE1C in the GSE8671 **(A)**, GSE21510 **(B)**, GSE9348 **(C)**, TCGA database **(D, E)** are shown. **(F)** Expression of PDE1C in pan-cancer. ACC, adrenocortical carcinoma; BLCA, bladder urothelial carcinoma; BRCA, breast invasive carcinoma; CESC, cervical and endocervical cancers; CHOL, cholangiocarcinoma; COAD, colorectal adenocarcinoma; DLBC, lymphoid neoplasm diffuse large B-cell lymphoma; ESCA, oesophageal carcinoma; GBM, glioblastoma multiforme; HNSC, head and neck squamous cell carcinoma; KICH, kidney chromophobe; KIRC, kidney renal clear cell carcinoma; KIRP, kidney renal papillary cell carcinoma; LAML, acute myeloid leukaemia; LGG, brain lower grade glioma; LIHC, liver hepatocellular; LUAD, lung adenocarcinoma; LUSC, lung squamous cell carcinoma; OV, ovarian serous cystadenocarcinoma; PAAD, pancreatic adenocarcinoma; PCPG, pheochromocytoma and paraganglioma; PRAD, prostate adenocarcinoma; READ, rectum adenocarcinoma; SARC, sarcoma; SKCM, skin cutaneous melanoma; STAD, stomach adenocarcinoma; TGCT, testicular germ cell tumour; THCA, thyroid carcinoma; TNYM, thymoma; UCEC, uterine corpus endometrial carcinoma; UCS, uterine carcinosarcoma. *P < 0.05

We performed an eQTL analysis in order to explore whether rs12538364 and mRNA expression of *PDE1C* are correlated. As shown in **Supplementary Figure 3C**, the T allele of the SNP rs12538364 was not associated with significantly lower levels of *PDE*1C mRNA expression in the TCGA database. There may be a lack of sufficient data. (*P* > 0.05, **Supplementary Figures 3C–D**). According to the dual-luciferase reporter assays conducted on DLD-1 and HCT116 cells, the C allele of rs12538364 had higher promoter activity than the T allele (*P* < 0.05, **Supplementary Figures 3E–F**). We discovered that in the GEO database (*P* = 2.50 × 10^-3^ in GSE8671, *P* = 1.81 × 10^-12^ in GSE21510, *P* = 1.93 × 10^-2^ in GSE9348), as shown in **Figures 2A–C**. In addition, our findings were also verified in the TCGA database (*P* = 2.56 × 10^-25^ in unpaired samples and *P* = 1.23×10^-3^in matched samples; **Figures 2D, E**). *PDE1C* was downregulated in multiple tumor tissues (**Figure 2F**)

### *PDE1C* is associated with immune cell infiltrations in colorectal cancer

**Figure 3:**
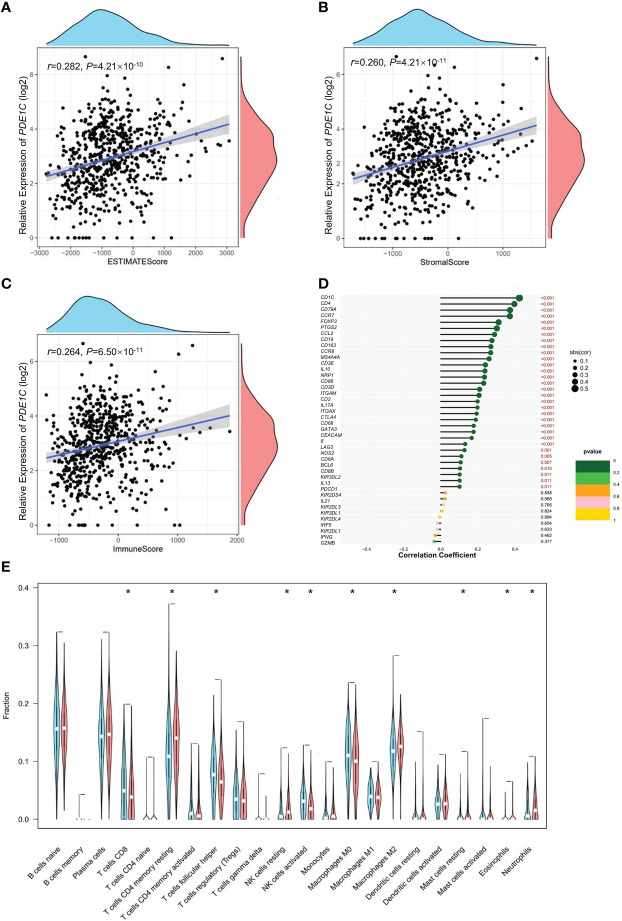
Immunity analysis of colorectal cancer. **(A)** Correlation analysis of *PDE1C* mRNA level expression level overall score of the tumor microenvironment. **(B)** Correlation analysis of *PDE1C* mRNA expression level and the tumor microenvironment stromal cell score. **(C)** Correlation analysis of *PDE1C* mRNA expression level and immune cell score in the tumor microenvironment. **(D)** Correlation analysis between *PDE1C* mRNA expression level and immune cell maker genes in the TCGA. **(E)** *PDE1C* high and low mRNA expression level group variance analysis with 22 immunity cells. *P < 0.05.

*PDE1C* is significantly correlated to immune/stromal/ESTIMATE scores in colorectal cancer. In CRC, the relationships between *PDE1C* expression and immunological, stromal, and ESTIMATE scores were investigated further. In colorectal cancer, increased *PDE1C* mRNA expression was association with higher ESTIMATE scores (*P* < 0.05, **Figure 3A**), stromal scores (*P* < 0.05, **Figure 3B**), and immune scores (*P* < 0.05, **Figure 3C**) in colorectal cancer. These findings indicated that *PDE1C* was distinctly related to the tumor immune microenvironment of colorectal cancer. In addition to this, we found only statistically significant relationship between immune cell score and colorectal cancer development (*P* < 0.05, **Supplementary Figure 4**). This means that the clinical phenotype, age and gender cannot effectively determine the immune infiltration status of colorectal cancer patients and cannot be used as one of the indicators for individualized treatment of colorectal cancer patients.

Using CIBERSORT (**Supplementary Figure 5**), we looked at 22 different kinds of tumor-infiltrating immune cells in the TCGA colorectal cancer cohort and examined the relationship between them. In every instance, we discovered that NK cells were the most abundant. Furthermore, the majority of immune cells compensate one another (*P* < 0.05, **Supplementary Figure 6**). We further analyzed the association between *PDE1C* mRNA expression level and 22 immune cell infiltrations for colorectal cancer. In **Figure 3D**, our data showed that *PDE1C* expression RNA-seq was significantly correlated with immunity cells including macrophages (M0, M2), mast cell resting and NK cell activated (*P* < 0.05). We investigated the correlation between *PDE1C* expression levels and 22 different kinds of tumor-infiltrating immune cells. Our findings revealed that high and low expression levels of *PDE1C* corrected CD4^+^ T cells, T cell follicular helper, NK cells activated, NK cells resting, macrophages (M0, M2), mast cell resting, eosinophils, and neutrophils (*P* < 0.05). Furthermore, *PDE1C* was shown to be highly related to practically all immune-related indicators (*P* < 0.05, **Figure 3E**).

### Immunity factors and clinical treatment

**Figure 4:**
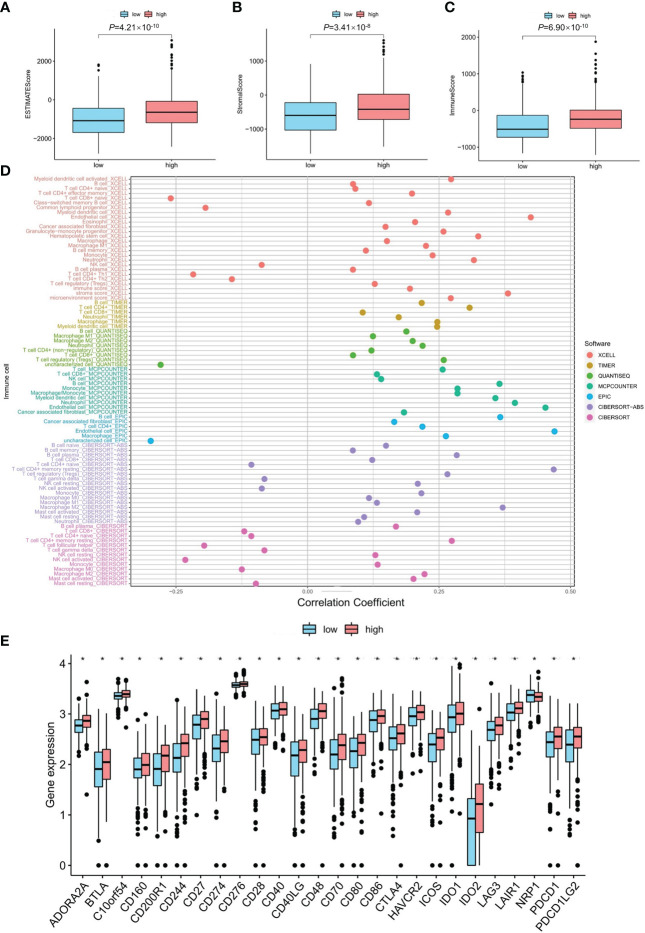
The investigation of tumor immune factors and immunotherapy. **(A–C)** The comparison of immune-related scores, estimate-related scores and stormal-related scores between low and high mRNA level of *PDE1C*. **(D)** The immune cell bubble between low- and high mRNA level of *PDE1C*. **(E)** The difference of 15 checkpoints expression between low- and high mRNA level of *PDE1C*. *P < 0.05.

The high mRNA expression level of the *PDE1C* group had a higher immune score and a higher ESTIMATE (microenvironment) score, signifying a different tumor microenvironment from the low mRNA expression level of the *PDE1C* group (*P* < 0.05, **Figures 4A–C**). Numerous immune cells were related to the *PDE1C* group of high mRNA expression level on several platforms, as shown in the immune cell bubble chart and document, including macrophage M1, T cell CD8^+^ naive at XCELL. At TIMER, B cell, T cell CD8^+^, and T cell CD4^+^ were detected; macrophage M1 was detected with QUANTISEQ; and B cell was detected at MCPcounter and EPIC (*P* < 0.05, **Figure 4D**). Most immune checkpoints were also more activated in the *PDE1C* group with high mRNA expression levels. (*P* < 0.05, **Figure 4E**). This means that we may choose relevant checkpoint inhibitors for colorectal cancer patients classified according to their high *PDE1C* mRNA expression levels (22).

## Discussion

An initial objective of the project was to identify genetic variants in the calcium signaling pathway gene-modified colorectal cancer through the tumor microenvironment. One interesting finding is that changing the rs12538364 T allele in *PDE1C* to the rs12538364 C allele dramatically increases the risk of colorectal cancer. Additionally, *PDE1C* may function as a guide for immunotherapy in colorectal cancer.

*PDE1C* rs12538364 is in an intron, which could affect gene expression in different ways. According to published research, histone modification enrichment may contribute to gene expression change (23). Previous research indicated that genetic variations alter the chromatin structure, affecting gene expression, which may result in carcinogenesis and tumor growth (24, 25). Stratification analyses of demographic data revealed that the rs12538364 T allele was a risk factor for colorectal cancer in practically all categories except those who smoked. The use of alcoholic beverages is a significant risk factor for colorectal cancer (23). We discovered that people who used alcohol had an increased chance of getting colorectal cancer than those who did not. In terms of clinicopathologic features, stratification analysis revealed that mutant alleles acted as risk factors in all colorectal cancer subgroups. Mutant RNA secondary structure stability decreased due to an increase in MFE. Aberrant post-transcriptional regulation and translation efficiency are possible consequences of this variation (26). Mutation of the intron region rs12538364 C site to the T site reduces the promoter activity of *PDE1C* in DLD1 and HCT116 cell lines. Hence, colorectal cancer may occur as a result.

*PDE1C* is a significant member of the phosphodiesterase 1 (PDE1) superfamily, which is a diesterase with dual substrates (cAMP and cGMP) and is a prospective target for the treatment of colorectal cancer (27). The binding of PDE1 to Ca^2+^/calmodulin (CaM) stimulates its enzymatic activity, resulting in the integration of Ca^2+^ and cyclic nucleotide-mediated signaling in a variety of illnesses (27). Glioblastoma proliferation and migration are associated with *PDE1C (* 28). Our findings indicate that *PDE1C* is associated with the risk of colorectal cancer.

Due to their capacity to detect and lyse altered cells, NK cells may help prevent the development of cancer. Increased intracellular Ca^2+^ concentrations are essential for effective NK cell activity and, consequently, for target death (29). Elevated [Ca^2+^] is one of the triggering signals for T cell activation induced by antigens (30). Interventions in ER Ca^2+^ signaling in CD^4+^ T cells have been postulated as a strategy for rehabilitating the tumor microenvironment’s immunological activity (15). Additionally, calcium signals have a direct function in controlling cancer cell death produced by cytotoxic T lymphocytes and natural killer cells (15). Additionally, our results indicated that *PDE1C* was significantly connected with immune-related marker genes (*P* < 0.05). As a result, *PDE1C* may be implicated in modulating immune-related pathways in the microenvironment of colorectal cancer.

Systemic therapy is the sole option for people who are unable to be treated surgically. Chemotherapeutics and target therapeutics are regularly used systemic therapies that typically result in treatment failure and adverse events (3, 31). It compels us to develop novel treatment techniques. Immunotherapy has altered the landscape of cancer treatment and accomplished a great deal. However, in the majority of malignancies, only one-third of patients react to checkpoint inhibitors (4). Immunotherapy resistance may also be attributed to a lack of previous immunity (32). As a result, we classified patients into two groups according to the expression of *PDE1C.* The two groups, as predicted, exhibited distinct immune microenvironments. The low mRNA expression levels of *PDE1C* was associated with an immunosuppressive TME. Simultaneously, the high mRNA expression levels of *PDE1C* had a greater number of CD8^+^ T cells that were strongly infiltrated, a more active function of inflammation promotion, a higher immune score, and a higher activity of PD-L1, CD27, and CD40 (21).

Our present study has several limitations. To begin with, there is a dearth of evidence showing eQTL between SNPs and low mRNA expression of the *PDE1C* in larger populations. Second, further data are required to confirm the association between the calcium signaling pathway gene polymorphism and the risk of colorectal cancer. The molecular mechanism through which polymorphisms in the calcium signaling pathway are connected with colorectal cancer development *via* the tumor microenvironment needs further data and biological experimentation. The rs12538364 under study is located in an intron of *PDE1C*. Although introns are noncoding regions of DNA where the vast majority (over 90%) of variants associated with complex phenotypes have been detected (33), they may be involved in the underlying pathogenic mechanism by regulating mRNA expression. These findings are likely driven by the top-hit SNP rs12538364 as suggested in our present study based on genetic association. Based on functional annotation from ENCODE data, rs12538364 is located in histone marks of H2AFZ, H3K27ac H3K27me3, H3K36me3, H3K4me3, and H3K4me2 and/or nearby DNase I hypersensitive sites (34). Given that the chromatin modifications of histone marks are vital to regulating gene expression and that DNase-sensitive sites tend to be clustered in transcriptional start regions, this evidence highlights the potential role of this SNP in regulating *PDE1C* expression. In the next study, we will verify at the molecular biology experimental level whether histone tagging affects *PDE1C* expression levels. We will perform ChIP-PCR experiments with antibodies against H2AFZ, H3K27ac H3K27me3, H3K36me3, H3K4me3, and H3K4me2 marks are associated with gene activation. We tested for the presence of these marks in HCT116 clones, one in which the C allele of rs12538364 was deleted and one in which the T allele of rs12538364 was deleted. We will select four colorectal cancer cell lines for knockdown as well as overexpression of *PDE1C* in order to construct stable transgenic cell lines. Subsequently, proliferation, invasion and metastasis cell cycle assays were performed. One of the cell lines was selected for multi-omics sequencing to find downstream to explore the mechanism. In parallel, we expect to collect 100 human colorectal cancer tissues for immunohistochemical staining. To verify the expression level of *PDE1C* in the tissues, we also extracted tissue RNA and tissue protein. To verify the expression of *PDE1C* at the molecular level.

Overall, we explore the relationship between polymorphisms in the calcium signaling pathway genes and colorectal cancer risk in the Chinese population. Due to the low expression of the *PDE1C* in colorectal cancer, *PDE1C* may be a potential therapeutic target and tumor biomarker for colorectal cancer. We investigate the mechanism by which genetic variants in the calcium signaling pathway genes contribute to colorectal cancer pathogenesis *via* the tumor microenvironment, and our findings provide a new perspective on the molecular mechanism underlying genetic variant and colorectal cancer risk.

## Data availability statement

Publicly available datasets were analyzed in this study. This data can be found here: TCGA (https://portal.gdc.cancer.gov/), GSEA (http://www.gseamsigdb.org/gsea/index.jsp).

## Ethics statement

The studies involving human participants were reviewed and approved by the First Affiliated Hospital of Nanjing Medical University, Nanjing, China. The patients/participants provided their written informed consent to participate in this study.

## Author contributions

J-YW and YS contributed equally to this study. J-YW and YS participated in the conceptualization. The project proposal design, project schedule, and original manuscript preparation were contributed by J-YW. The examination and editing of the manuscript are carried out by J-YW, C-ZH, YS, Z-LW, H-QZ, and ZF. ZF assisted with project administration and funding acquisition. All authors contributed to the article and approved the submitted version.

## References

[1] Siegel RL Miller KD Fuchs HE Jemal A. Cancer statistics, 2021. CA Cancer J Clin (2021) 71(1):7–33. doi: 10.3322/caac.21654 33433946

[2] Brenner H Kloor M Pox CP. Colorectal cancer. Lancet (9927) 2014:1490–502:383. doi: 10.1016/S0140-6736(13)61649-9 24225001

[3] Ganesh K Stadler ZK Cercek A Mendelsohn RB Shia J Segal NH. Immunotherapy in colorectal cancer: rationale, challenges and potential. Nat Rev Gastroenterol Hepatol (2019) 16(6):361–75. doi: 10.1038/s41575-019-0126-x PMC7295073 30886395

[4] Tang R Xu J Zhang B Liu J Liang C Hua J. Ferroptosis, necroptosis, and pyroptosis in anticancer immunity. J Hematol Oncol (2020) 13(1):110. doi: 10.1186/s13045-020-00946-7 32778143 PMC7418434

[5] Hughes LAE Simons C van den Brandt PA van Engeland M Weijenberg MP. Lifestyle, diet, and colorectal cancer risk according to (Epi)genetic instability: Current evidence and future directions of molecular pathological epidemiology. Curr Colorectal Cancer Rep (2017) 13(6):455–69. doi: 10.1007/s11888-017-0395-0 PMC5725509 29249914

[6] Buniello A MacArthur JAL Cerezo M Harris LW Hayhurst J Malangone C. The NHGRI-EBI GWAS catalog of published genome-wide association studies, targeted arrays and summary statistics 2019. Nucleic Acids Res (2019) 47(D1):D1005–D12. doi: 10.1093/nar/gky1120 PMC6323933 30445434

[7] Monteith GR McAndrew D Faddy HM Roberts-Thomson SJ. Calcium and cancer: targeting Ca2+ transport. Nat Rev Cancer (2007) 7(7):519–30. doi: 10.1038/nrc2171 17585332

[8] Roderick HL Cook SJ. Ca2+ signalling checkpoints in cancer: remodelling Ca2+ for cancer cell proliferation and survival. Nat Rev Cancer (2008) 8(5):361–75. doi: 10.1038/nrc2374 18432251

[9] Humeau J Bravo-San Pedro JM Vitale I Nunez L Villalobos C Kroemer G. Calcium signaling and cell cycle: Progression or death. Cell Calcium (2018) 70:3–15. doi: 10.1016/j.ceca.2017.07.006 28801101

[10] Clapham DE. Calcium signaling. Cell. (2007) 131(6):1047–58. doi: 10.1016/j.cell.2007.11.028 18083096

[11] Hanahan D Weinberg RA. Hallmarks of cancer: the next generation. Cell (2011) 144(5):646–74. doi: 10.1016/j.cell.2011.02.013 21376230

[12] Keum N Giovannucci E. Global burden of colorectal cancer: emerging trends, risk factors and prevention strategies. Nat Rev Gastroenterol Hepatol (2019) 16(12):713–32. doi: 10.1038/s41575-019-0189-8 31455888

[13] Monteith GR Davis FM Roberts-Thomson SJ. Calcium channels and pumps in cancer: changes and consequences. J Biol Chem (2012) 287(38):31666–73. doi: 10.1074/jbc.R112.343061 PMC3442501 22822055

[14] Tsavaler L Shapero MH Morkowski S Laus R. Trp-p8, a novel prostate-specific gene, is up-regulated in prostate cancer and other malignancies and shares high homology with transient receptor potential calcium channel proteins. Cancer Res (2001) 61(9):3760–9. 11325849

[15] Monteith GR Prevarskaya N Roberts-Thomson SJ. The calcium-cancer signalling nexus. Nat Rev Cancer. (2017) 17(6):367–80. doi: 10.1038/nrc.2017.18 28386091

[16] Meng Y Li S Gu D Xu K Du M Zhu L. Genetic variants in m6A modification genes are associated with colorectal cancer risk. Carcinogenesis. (2020) 41(1):8–17. doi: 10.1093/carcin/bgz165 31579913

[17] Tsukamoto S Ishikawa T Iida S Ishiguro M Mogushi K Mizushima H. Clinical significance of osteoprotegerin expression in human colorectal cancer. Clin Cancer Res (2011) 17(8):2444–50. doi: 10.1158/1078-0432.CCR-10-2884 21270110

[18] Sabates-Bellver J van der Flier LG de Palo M Cattaneo E Maake C Rehrauer H. Transcriptome profile of human colorectal adenomas. Mol Cancer Res (2007) 5(12):1263–75. doi: 10.1158/1541-7786.MCR-07-0267 18171984

[19] Hong Y Downey T Eu KW Koh PK Cheah PY. A ‘metastasis-prone’ signature for early-stage mismatch-repair proficient sporadic colorectal cancer patients and its implications for possible therapeutics. Clin Exp Metastasis. (2010) 27(2):83–90. doi: 10.1007/s10585-010-9305-4 20143136

[20] Newman AM Liu CL Green MR Gentles AJ Feng W Xu Y. Robust enumeration of cell subsets from tissue expression profiles. Nat Methods (2015) 12(5):453–7. doi: 10.1038/nmeth.3337 PMC4739640 25822800

[21] Zheng Y Tian H Zhou Z Xiao C Liu H Liu Y. A novel immune-related prognostic model for response to immunotherapy and survival in patients with lung adenocarcinoma. Front Cell Dev Biol (2021) 9:651406. doi: 10.3389/fcell.2021.651406 33816503 PMC8017122

[22] Johdi NA Sukor NF. Colorectal cancer immunotherapy: Options and strategies. Front Immunol (2020) 11:1624. doi: 10.3389/fimmu.2020.01624 33042104 PMC7530194

[23] Fagunwa IO Loughrey MB Coleman HG. Alcohol, smoking and the risk of premalignant and malignant colorectal neoplasms. Best Pract Res Clin Gastroenterol (2017) 31(5):561–8. doi: 10.1016/j.bpg.2017.09.012 29195676

[24] Nacev BA Jones KB Intlekofer AM Yu JSE Allis CD Tap WD. The epigenomics of sarcoma. Nat Rev Cancer. (2020) 20(10):608–23. doi: 10.1038/s41568-020-0288-4 PMC8380451 32782366

[25] Spielmann M Lupiáñez DG Mundlos S. Structural variation in the 3D genome. Nat Rev Genet (2018) 19(7):453–67. doi: 10.1038/s41576-018-0007-0 29692413

[26] He F Wei R Zhou Z Huang L Wang Y Tang J. Integrative analysis of somatic mutations in non-coding regions altering RNA secondary structures in cancer genomes. Sci Rep (2019) 9(1):8205. doi: 10.1038/s41598-019-44489-5 31160636 PMC6546760

[27] Samidurai A Xi L Das A Iness AN Vigneshwar NG Li PL. Role of phosphodiesterase 1 in the pathophysiology of diseases and potential therapeutic opportunities. Pharmacol Ther (2021) 226:107858. doi: 10.1016/j.pharmthera.2021.107858 33895190 PMC8815087

[28] Rowther FB Wei W Dawson TP Ashton K Singh A Madiesse-Timchou MP. Cyclic nucleotide phosphodiesterase-1C (PDE1C) drives cell proliferation, migration and invasion in glioblastoma multiforme cells *in vitro* . Mol Carcinog (2016) 55(3):268–79. doi: 10.1002/mc.22276 25620587

[29] Schwarz EC Qu B Hoth M. Calcium, cancer and killing: the role of calcium in killing cancer cells by cytotoxic T lymphocytes and natural killer cells. Biochim Biophys Acta (2013) 1833(7):1603–11. doi: 10.1016/j.bbamcr.2012.11.016 23220009

[30] Feske S Giltnane J Dolmetsch R Staudt LM Rao A. Gene regulation mediated by calcium signals in T lymphocytes. Nat Immunol (2001) 2(4):316–24. doi: 10.1038/86318 11276202

[31] Feng M Zhao Z Yang M Ji J Zhu D. T-Cell-based immunotherapy in colorectal cancer. Cancer Lett (2021) 498:201–9. doi: 10.1016/j.canlet.2020.10.040 33129958

[32] Galon J Bruni D. Approaches to treat immune hot, altered and cold tumours with combination immunotherapies. Nat Rev Drug Discov (2019) 18(3):197–218. doi: 10.1038/s41573-018-0007-y 30610226

[33] Maurano MT Humbert R Rynes E Thurman RE Haugen E Wang H. Systematic localization of common disease-associated variation in regulatory DNA. Science. (2012) 337(6099):1190–5. doi: 10.1126/science.1222794 PMC3771521 22955828

[34] Yavartanoo M Choi JK. ENCODE: A sourcebook of epigenomes and chromatin language. Genomics Inform. (2013) 11(1):2–6. doi: 10.5808/GI.2013.11.1.2 23613676 PMC3630381

