# Supplementary figures and images for: Genetic variants in the calcium signaling pathway participate in the pathogenesis of colorectal cancer through the tumor microenvironment
Source: Front Oncol. 2023 Feb 7;13:992326. doi: 10.3389/fonc.2023.992326 (PMC9941622; doi:10.3389/fonc.2023.992326)

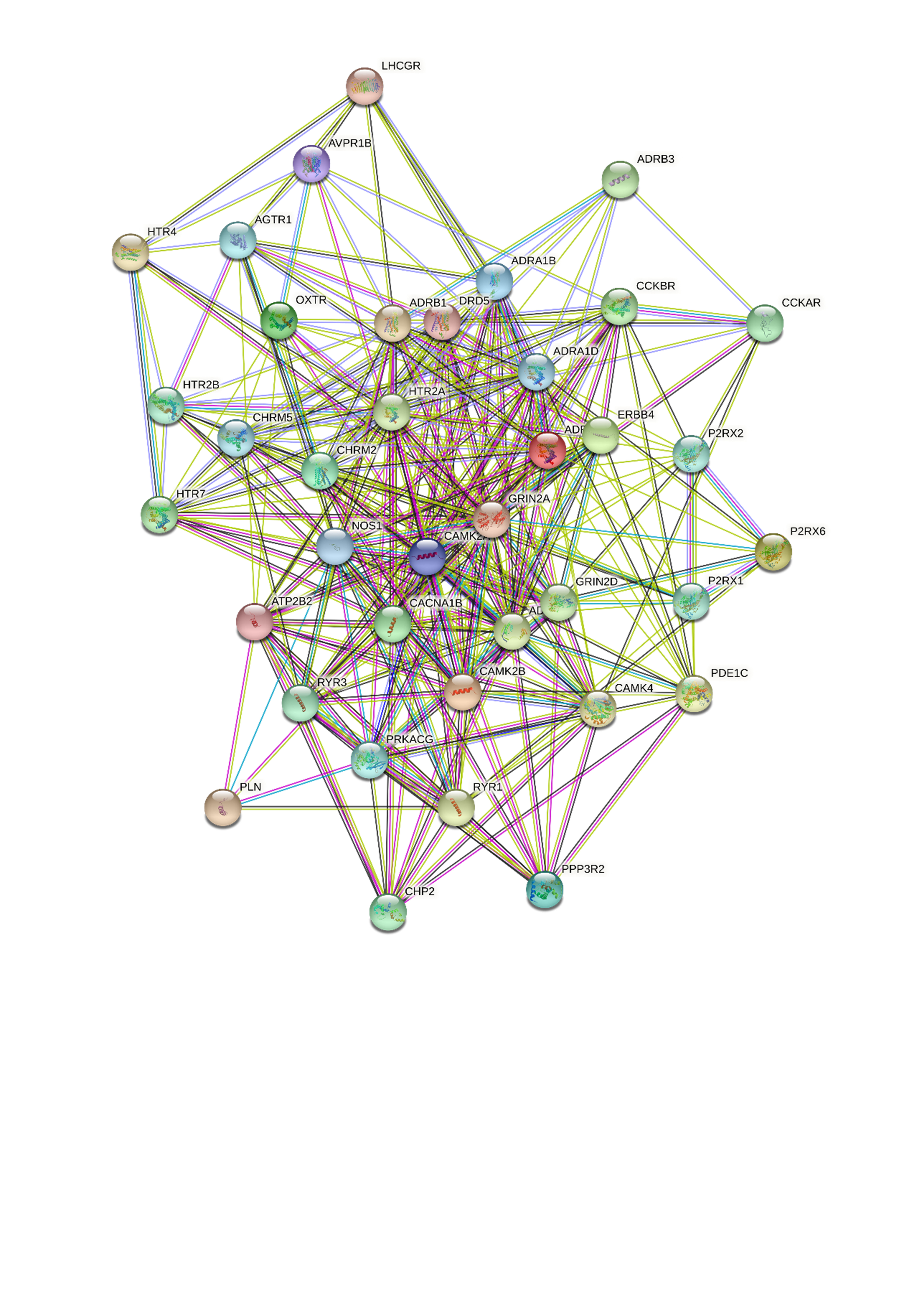

Supplement: Supplementary file 1 [file Image_1.tif]

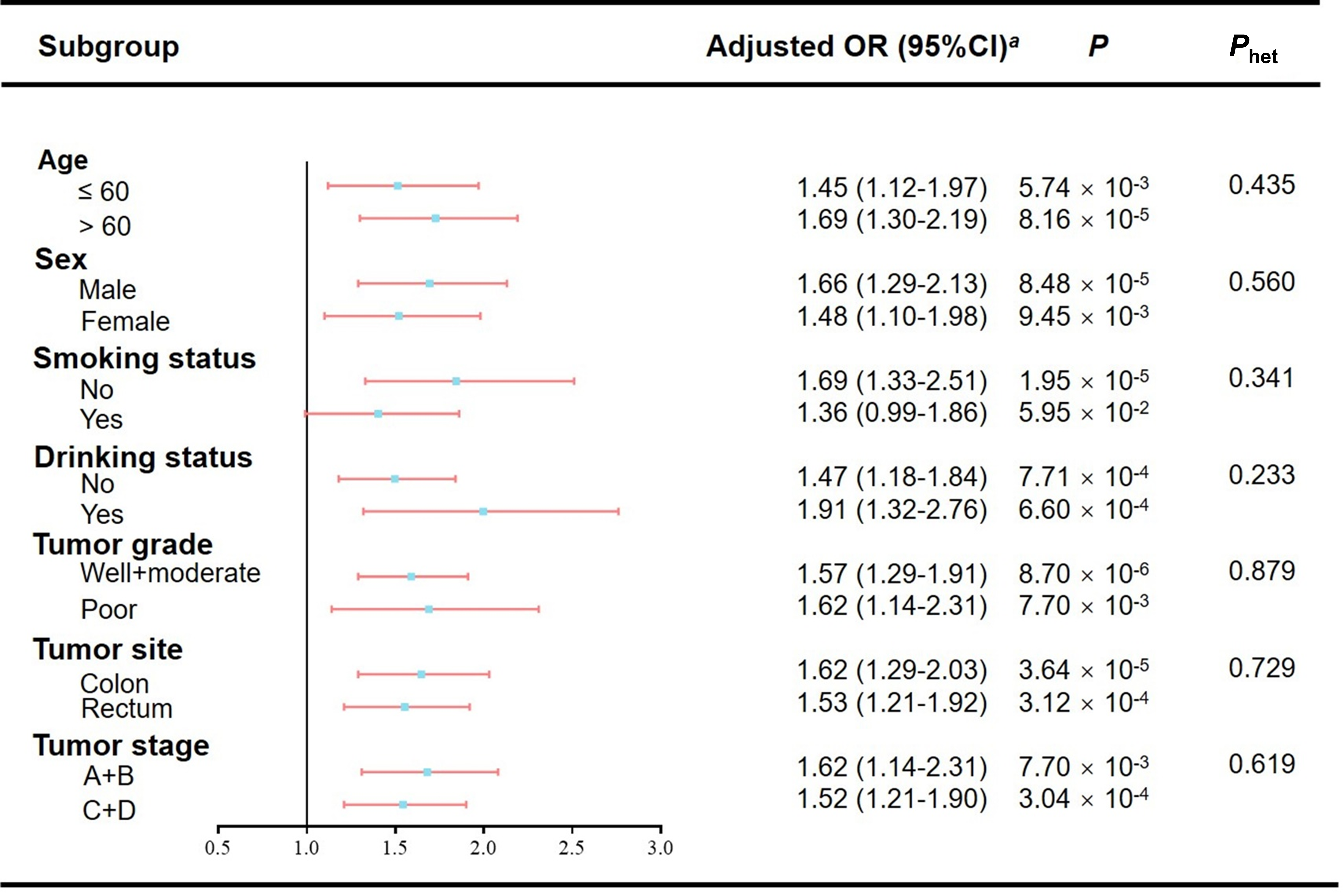

Supplement: Supplementary file 2 [file Image_2.tif]

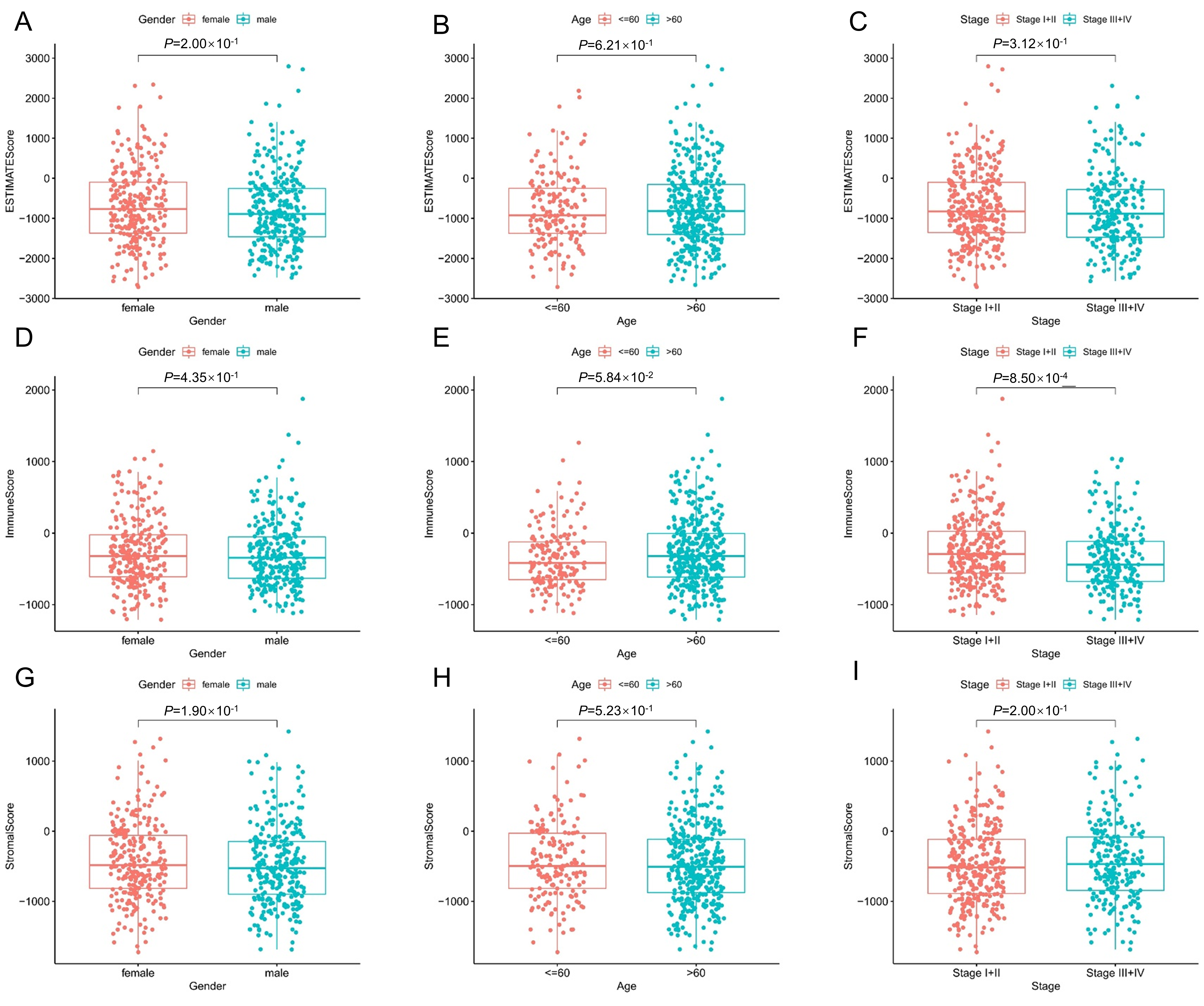

Supplement: Supplementary file 3 [file Image_3.tif]

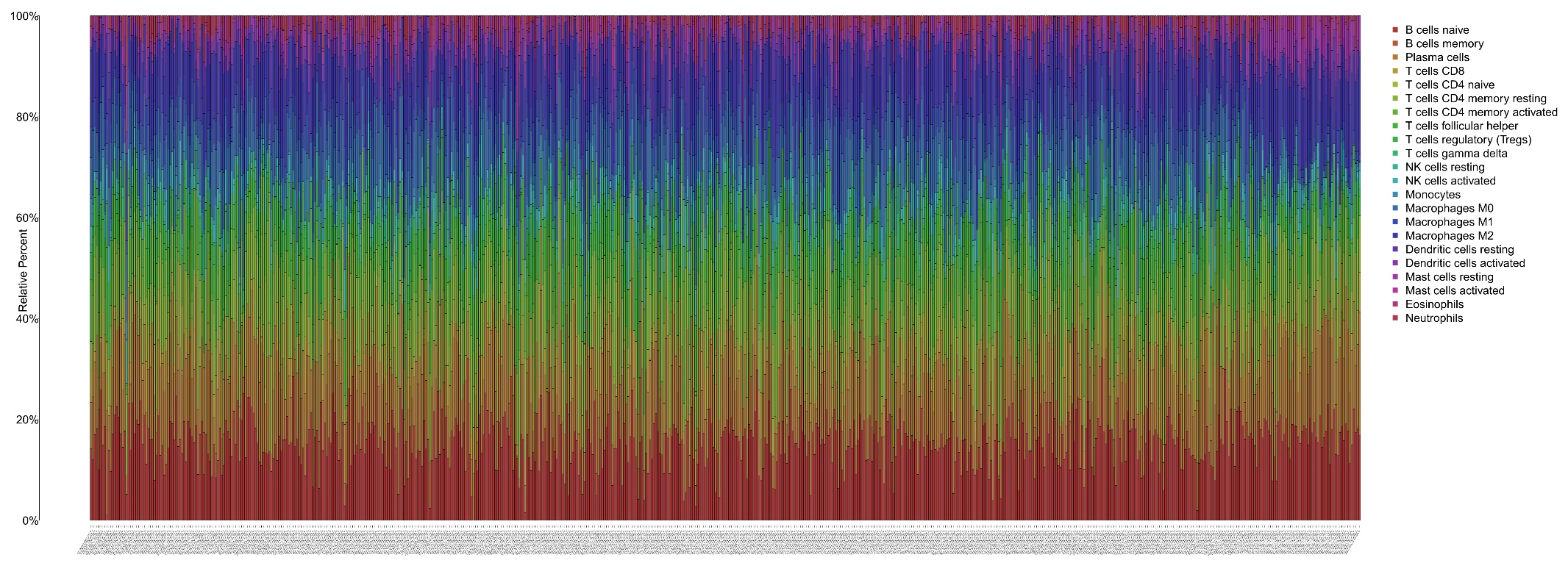

Supplement: Supplementary file 4 [file Image_4.tif]

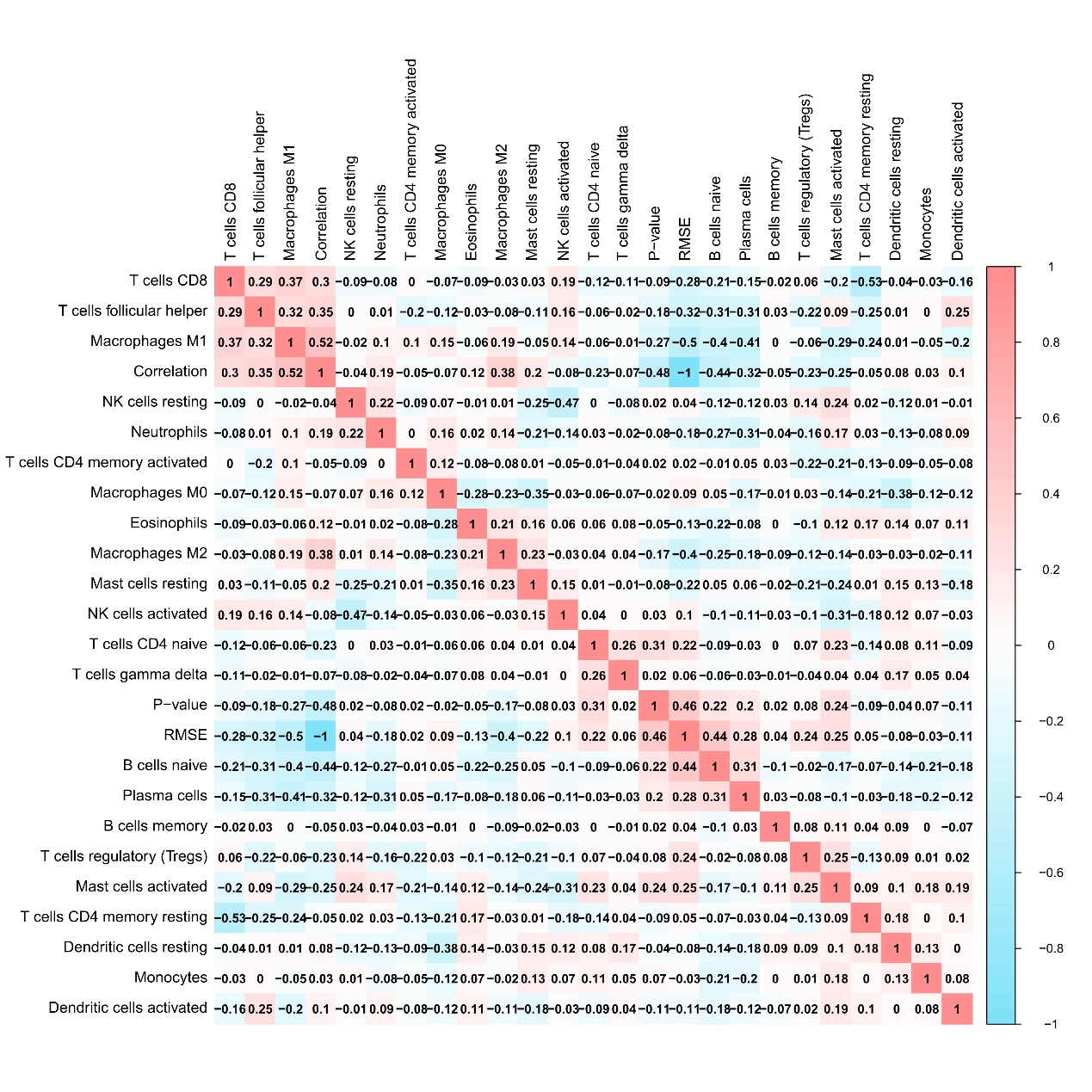

Supplement: Supplementary file 5 [file Image_5.tif]

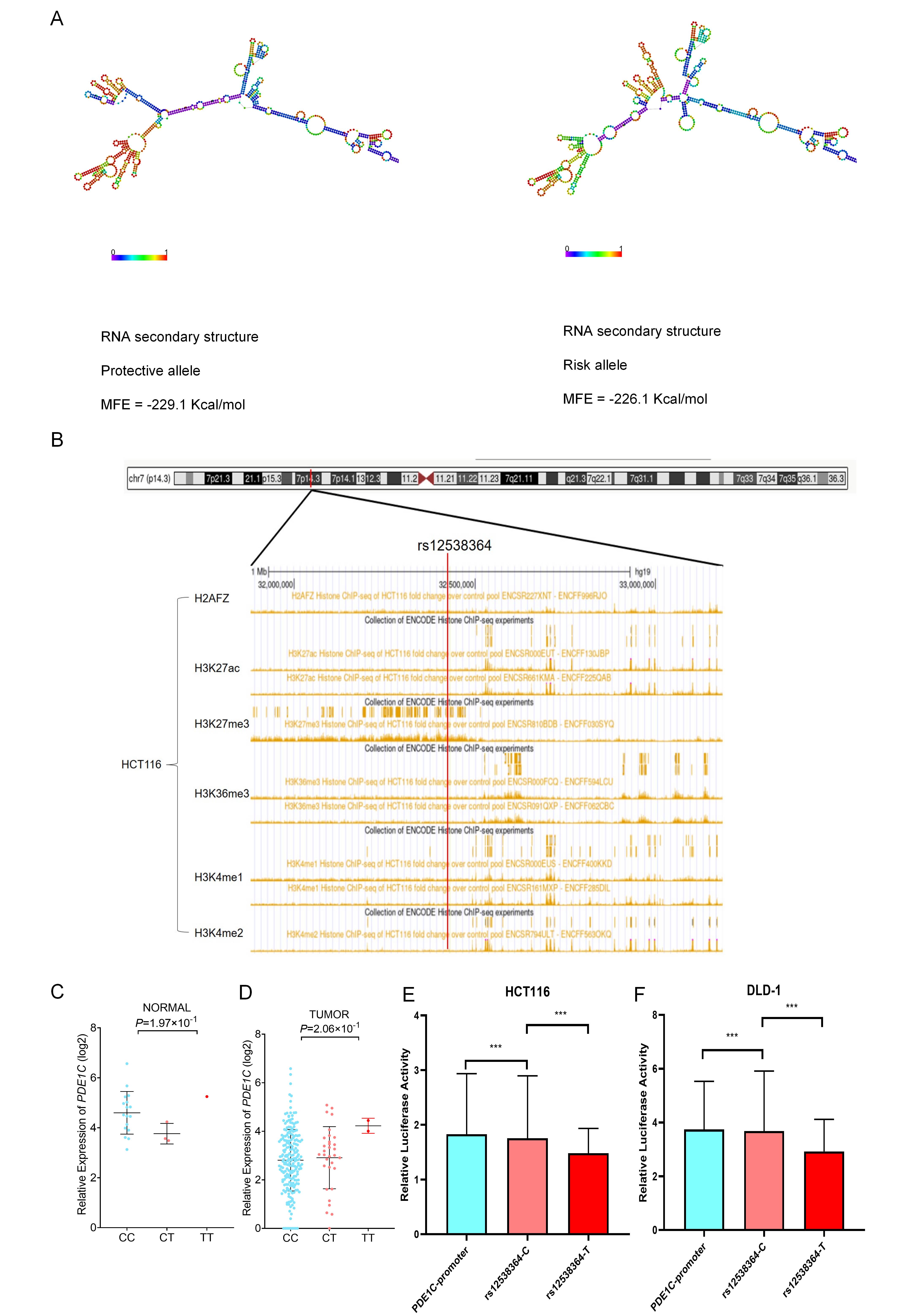

Supplement: Supplementary file 6 [file Image_6.jpeg]
