# Supplementary material for: Genetic variants in the calcium signaling pathway participate in the pathogenesis of colorectal cancer through the tumor microenvironment
Source: Front Oncol. 2023 Feb 7;13:992326. doi: 10.3389/fonc.2023.992326 (PMC9941622; doi:10.3389/fonc.2023.992326)
Supplement: Supplementary file 7 [file Table_1.docx]

**Supplementary Table 1. Differential expression of 38 Calcium signaling pathway genes in TCGA and GEO.**

| Gene | TCGA | | GSE201510 | |
| --- | --- | --- | --- | --- |
|  | Log_2_(Fold change) | *P*-value | Log_2_(Fold change) | *P*-value |
| *ADCY2* | 0.63 | 2.10 × 10^-14^ | 0.89 | 1.37 × 10^-39^ |
| *ADRA1A* | 0.25 | 2.30 × 10^-19^ | 0.84 | 1.60 × 10^-56^ |
| *ADRA1B* | 0.57 | 4.00 × 10^-23^ | 0.83 | 3.83 × 10^-54^ |
| *ADRA1D* | 0.65 | 8.70 × 10^-6^ | 0.88 | 8.49 × 10^-36^ |
| *ADRB1* | 0.63 | 1.80 × 10^-43^ | 0.89 | 4.93 × 10^-03^ |
| *ADRB3* | 0.27 | 2.10 × 10^-20^ | 0.97 | 1.34 × 10^-7^ |
| *AGTR1* | 0.37 | 1.10 × 10^-22^ | 0.82 | 4.92 × 10^-7^ |
| *ATP2B2* | 0.31 | 1.70 × 10^-24^ | 0.87 | 1.77 × 10^-46^ |
| *AVPR1B* | 0.11 | 2.20 × 10^-21^ | 0.93 | 2.61 × 10^-34^ |
| *CACNA1B* | 0.48 | 1.80 × 10^-16^ | 0.93 | 2.23 × 10^-4^ |
| *CAMK2A* | 0.50 | 1.10 × 10^-20^ | 0.90 | 1.36 × 10^-33^ |
| *CAMK2B* | 0.51 | 6.10 × 10^-20^ | 0.86 | 1.23 × 10^-45^ |
| *CAMK4* | 0.63 | 5.60 × 10^-31^ | 0.92 | 2.87 × 10^-12^ |
| *CCKAR* | 0.35 | 5.30 × 10^-3^ | 0.97 | 7.86 × 10^-06^ |
| *CCKBR* | 0.57 | 1.04 × 10^-2^ | 0.93 | 9.30 × 10^-14^ |
| *CHP2* | 0.60 | 3.70 × 10^-66^ | 0.73 | 6.29 × 10^-16^ |
| *CHRM2* | 0.25 | 1.70 × 10^-15^ | 0.91 | 4.72 × 10^-19^ |
| *CHRM5* | 0.47 | 4.50 × 10^-16^ | 0.91 | 2.16 × 10^-25^ |
| *DRD5* | 0.33 | 3.90 × 10^-06^ | 0.80 | 6.22 × 10^-55^ |
| *ERBB4* | 0.24 | 9.90 × 10^-15^ | 0.91 | 4.61 × 10^-27^ |
| *GRIN2A* | 0.29 | 1.10 × 10^-35^ | 0.89 | 1.73 × 10^-30^ |
| *GRIN2D* | 2.17 | 2.80 × 10^-47^ | 1.12 | 6.14 × 10^-10^ |
| *HTR2A* | 0.62 | 2.00 × 10^-10^ | 0.90 | 9.16 × 10^-30^ |
| *HTR2B* | 0.63 | 1.20 × 10^-13^ | 0.90 | 1.03 × 10^-4^ |
| *HTR4* | 0.40 | 1.60 × 10^-58^ | 0.77 | 1.61 × 10^-18^ |
| *HTR7* | 0.45 | 7.30 × 10^-39^ | 0.89 | 1.64 × 10^-36^ |
| *LHCGR* | 0.36 | 8.59 × 10^-3^ | 0.89 | 1.49 × 10^-40^ |
| *NOS1* | 0.30 | 1.20 × 10^-10^ | 0.86 | 2.56 × 10^-31^ |
| *OXTR* | 1.77 | 2.70 × 10^-42^ | 1.18 | 1.94 × 10^-11^ |
| *P2RX1* | 0.63 | 4.20 × 10^-61^ | 0.91 | 1.64 × 10^-37^ |
| *P2RX2* | 0.17 | 3.90 × 10^-26^ | 0.88 | 2.61 × 10^-32^ |
| *P2RX6* | 0.62 | 1.30 × 10^-14^ | 0.87 | 5.21 × 10^-51^ |
| *PDE1C* | 0.62 | 5.10 × 10^-25^ | 0.93 | 1.81 × 10^-22^ |
| *PLN* | 0.61 | 2.20 × 10^-18^ | 0.84 | 2.31 × 10^-4^ |
| *PPP3R2* | 0.28 | 4.90 × 10^-5^ | 0.89 | 3.43 × 10^-39^ |
| *PRKACG* | 0.59 | 7.62 × 10^-3^ | 0.93 | 9.61 × 10^-24^ |
| *RYR1* | 0.66 | 5.90 × 10^-21^ | 0.93 | 1.28 × 10^-15^ |
| *RYR3* | 0.53 | 1.40 × 10^-15^ | 0.86 | 1.44 × 10^-9^ |
