# Supplementary material for: Genetic variants in the calcium signaling pathway participate in the pathogenesis of colorectal cancer through the tumor microenvironment
Source: Front Oncol. 2023 Feb 7;13:992326. doi: 10.3389/fonc.2023.992326 (PMC9941622; doi:10.3389/fonc.2023.992326)
Supplement: Supplementary file 8 [file Table_2.docx]

**Supplementary Table 2. Associations between 2 selected SNPs and colorectal cancer risk in the Chinese population.**

| SNPs | Gene | Chr | Position(hg19) | Call rate | Allele^a^ | MAF | | *P*_(HWE)_^b^ | Adjusted OR (95%CI)^c^ | *P*^c^ | *P*_FDR_ |
| --- | --- | --- | --- | --- | --- | --- | --- | --- | --- | --- | --- |
|  |  |  |  |  |  | Cases | Controls |  |  |  |  |
| rs34786964 | *AVPR1B* | 1 | 32350106 | 0.95 | T/C | 0.12 | 0.09 | 0.18 | 1.35 (1.12-1.61) | 1.36 × 10^-3^ | 0.183 |
| rs13398000 | *ERBB4* | 2 | 213179162 | 0.98 | A/G | 0.15 | 0.12 | 0.70 | 1.22 (1.04-1.44) | 1.47 × 10^-2^ | 0.406 |
| rs1394793 | *ERBB4* | 2 | 213178061 | 0.96 | T/G | 0.19 | 0.16 | 0.53 | 1.25 (1.08-1.45) | 3.57 × 10^-3^ | 0.241 |
| rs1505361 | *RYR3* | 2 | 33896190 | 1.00 | A/G | 0.09 | 0.07 | 0.31 | 1.25 (1.02-1.53) | 3.38 × 10^-2^ | 0.488 |
| rs16848730 | *RYR3* | 2 | 33788557 | 0.99 | G/A | 0.09 | 0.07 | 0.67 | 1.31 (1.06-1.61) | 1.29 × 10^-2^ | 0.411 |
| rs1836735 | *ERBB4* | 2 | 212868623 | 0.99 | A/G | 0.35 | 0.32 | 0.23 | 1.15 (1.02-1.29) | 2.49 × 10^-2^ | 0.432 |
| rs1879637 | *RYR1* | 2 | 38992354 | 1.00 | C/T | 0.06 | 0.08 | 0.57 | 0.76 (0.61-0.95) | 1.46 × 10^-2^ | 0.413 |
| rs2289086 | *GRIN2A* | 2 | 10192777 | 0.99 | C/T | 0.44 | 0.42 | 0.17 | 1.12 (1.00-1.26) | 4.23 × 10^-2^ | 0.509 |
| rs34432856 | *HTR4* | 2 | 148030960 | 0.95 | A/C | 0.06 | 0.08 | 0.72 | 0.71 (0.57-0.89) | 2.49 × 10^-3^ | 0.189 |
| rs67044368 | *RYR3* | 2 | 33887513 | 0.96 | C/T | 0.12 | 0.10 | 0.07 | 1.29 (1.07-1.54) | 6.55 × 10^-3^ | 0.318 |
| rs6738323 | *PDE1C* | 2 | 32254013 | 0.96 | G/A | 0.46 | 0.49 | 0.58 | 0.89 (0.80-1.00) | 4.19 × 10^-2^ | 0.509 |
| rs1042778 | *OXTR* | 3 | 8794544 | 1.00 | T/G | 0.07 | 0.09 | 0.86 | 0.78 (0.63-0.97) | 2.28 × 10^-2^ | 0.440 |
| rs10510402 | *ATP2B2* | 3 | 10594177 | 0.99 | T/G | 0.18 | 0.16 | 0.47 | 1.18 (1.02-1.37) | 2.85 × 10^-2^ | 0.474 |
| rs2268492 | *CHRM2* | 3 | 136676569 | 0.97 | T/C | 0.15 | 0.13 | 0.62 | 1.21 (1.03-1.42) | 2.40 × 10^-2^ | 0.448 |
| rs4234503 | *ATP2B2* | 3 | 32397447 | 0.99 | T/C | 0.27 | 0.24 | 0.45 | 1.15 (1.01-1.31) | 3.23 × 10^-2^ | 0.497 |
| rs4684713 | *ADRA1A* | 3 | 26679577 | 0.97 | T/A | 0.13 | 0.11 | 1.00 | 1.20 (1.01-1.43) | 3.80 × 10^-2^ | 0.496 |
| rs749477 | *CAMK4* | 3 | 110652926 | 1.00 | A/G | 0.16 | 0.18 | 0.71 | 0.85 (0.73-0.99) | 3.19 × 10^-2^ | 0.503 |
| rs78409155 | *ADRA1B* | 3 | 159342740 | 0.97 | A/G | 0.07 | 0.09 | 0.86 | 0.81 (0.65-1.00) | 4.67 × 10^-2^ | 0.515 |
| rs79532153 | *RYR3* | 3 | 33998473 | 0.96 | A/G | 0.07 | 0.05 | 0.09 | 1.36 (1.08-1.72) | 9.82 × 10^-3^ | 0.362 |
| rs10058828 | *HTR4* | 5 | 147855791 | 0.98 | T/C | 0.07 | 0.09 | 0.20 | 0.78 (0.64-0.95) | 1.37 × 10^-2^ | 0.416 |
| rs10463293 | *CAMK2A* | 5 | 149636072 | 0.96 | T/G | 0.36 | 0.39 | 0.26 | 0.88 (0.79-0.99) | 3.60 × 10^-2^ | 0.491 |
| rs114941638 | *CAMK2A* | 5 | 149633579 | 0.96 | T/C | 0.08 | 0.06 | 0.13 | 1.44 (1.15-1.79) | 1.41 × 10^-3^ | 0.171 |
| rs13156542 | *HTR4* | 5 | 147836608 | 0.99 | T/C | 0.19 | 0.16 | 0.92 | 1.22 (1.06-1.42) | 7.51 × 10^-3^ | 0.326 |
| rs1500272 | *CAMK2A* | 5 | 149609971 | 1.00 | T/C | 0.09 | 0.10 | 0.77 | 0.81 (0.66-0.98) | 3.02 × 10^-2^ | 0.496 |
| rs1549913 | *ERBB4* | 5 | 213375865 | 0.96 | G/A | 0.43 | 0.40 | 0.64 | 1.15 (1.02-1.29) | 2.00 × 10^-2^ | 0.434 |
| rs1816066 | *GRIN2A* | 5 | 10060107 | 0.98 | A/G | 0.37 | 0.34 | 0.71 | 1.13 (1.01-1.27) | 4.18 × 10^-2^ | 0.512 |
| rs26739 | *PRKCG* | 5 | 54388478 | 1.00 | A/G | 0.50 | 0.47 | 0.55 | 1.12 (1.01-1.26) | 3.92 × 10^-2^ | 0.506 |
| rs35603339 | *CAMK2A* | 5 | 149625086 | 0.98 | A/C | 0.42 | 0.39 | 0.95 | 1.13 (1.00-1.27) | 4.55 × 10^-2^ | 0.526 |
| rs3756578 | *RYR3* | 5 | 34065523 | 0.96 | T/C | 0.44 | 0.47 | 0.20 | 0.88 (0.78-0.98) | 2.50 × 10^-2^ | 0.429 |
| rs3896275 | *ATP2B2* | 5 | 10617556 | 1.00 | T/C | 0.35 | 0.32 | 0.62 | 1.13 (1.01-1.27) | 3.66 × 10^-2^ | 0.495 |
| rs76322090 | *CAMK4* | 5 | 32397632 | 0.96 | T/C | 0.15 | 0.13 | 0.61 | 1.21 (1.02-1.44) | 2.55 × 10^-2^ | 0.429 |
| rs78938556 | *RYR3* | 5 | 34006773 | 0.97 | A/C | 0.12 | 0.09 | 0.32 | 1.28 (1.07-1.54) | 7.73 × 10^-3^ | 0.324 |
| rs79569615 | *NOS1* | 5 | 117689922 | 0.99 | T/C | 0.38 | 0.34 | 0.67 | 1.16 (1.03-1.30) | 1.38 × 10^-2^ | 0.410 |
| rs10225215 | *CHRM2* | 7 | 136630811 | 1.00 | A/C | 0.43 | 0.47 | 0.30 | 0.87 (0.78-0.97) | 1.53 × 10^-2^ | 0.413 |
| rs10239671 | *PDE1C* | 7 | 31794739 | 1.00 | C/T | 0.47 | 0.49 | 0.06 | 0.89 (0.79-1.00) | 4.58 × 10^-2^ | 0.525 |
| rs10486503 | *PDE1C* | 7 | 32248909 | 0.99 | T/G | 0.17 | 0.14 | 0.12 | 1.20 (1.03-1.40) | 1.73 × 10^-2^ | 0.438 |
| rs114853905 | *PDE1C* | 7 | 31886170 | 0.96 | T/A | 0.11 | 0.09 | 0.30 | 1.26 (1.04-1.52) | 1.88 × 10^-2^ | 0.431 |
| rs11760533 | *PDE1C* | 7 | 32128604 | 0.96 | A/T | 0.33 | 0.30 | 0.31 | 1.19 (1.05-1.35) | 5.38 × 10^-3^ | 0.297 |
| rs11761910 | *PDE1C* | 7 | 32134137 | 1.00 | A/G | 0.43 | 0.47 | 0.17 | 0.87 (0.78-0.98) | 1.76 × 10^-2^ | 0.428 |
| rs12536248 | *PDE1C* | 7 | 32369295 | 0.99 | G/A | 0.17 | 0.14 | 0.31 | 1.22 (1.05-1.43) | 9.90 × 10^-3^ | 0.354 |
| rs12538364 | *PDE1C* | 7 | 32429487 | 0.99 | T/C | 0.12 | 0.08 | 0.85 | 1.57 (1.30-1.91) | 3.07 × 10^-6^ | 0.004 |
| rs215742 | *OXTR* | 7 | 8800671 | 1.00 | A/G | 0.11 | 0.09 | 0.10 | 1.29 (1.08-1.55) | 5.85 × 10^-3^ | 0.309 |
| rs215747 | *ERBB4* | 7 | 212285102 | 1.00 | T/C | 0.34 | 0.31 | 0.25 | 1.17 (1.04-1.32) | 9.35 × 10^-3^ | 0.355 |
| rs2350786 | *GRIN2A* | 7 | 10069080 | 1.00 | G/A | 0.38 | 0.41 | 0.46 | 0.87 (0.78-0.98) | 1.66 × 10^-2^ | 0.429 |
| rs324626 | *AVPR1B* | 7 | 206222741 | 0.96 | C/G | 0.35 | 0.32 | 0.85 | 1.14 (1.01-1.29) | 3.19 × 10^-2^ | 0.497 |
| rs36199865 | *PDE1C* | 7 | 31891441 | 0.97 | G/C | 0.20 | 0.17 | 0.70 | 1.17 (1.01-1.36) | 3.37 × 10^-2^ | 0.493 |
| rs3801348 | *GRIN2A* | 7 | 10070894 | 0.97 | T/C | 0.43 | 0.40 | 0.08 | 1.14 (1.02-1.29) | 2.40 × 10^-2^ | 0.436 |
| rs4723146 | *RYR3* | 7 | 33967348 | 1.00 | C/T | 0.28 | 0.25 | 0.77 | 1.15 (1.01-1.30) | 3.34 × 10^-2^ | 0.494 |
| rs62457501 | *ERBB4* | 7 | 212300960 | 0.99 | G/A | 0.05 | 0.07 | 1.00 | 0.77 (0.61-0.98) | 3.13 × 10^-2^ | 0.507 |
| rs6966630 | *CHRM2* | 7 | 136626977 | 1.00 | C/T | 0.37 | 0.34 | 0.08 | 1.13 (1.01-1.27) | 3.15 × 10^-2^ | 0.504 |
| rs73158750 | *RYR3* | 7 | 33928884 | 0.97 | A/C | 0.37 | 0.39 | 0.73 | 0.89 (0.79-1.00) | 4.12 × 10^-2^ | 0.516 |
| rs73306564 | *PDE1C* | 7 | 32431245 | 0.99 | T/C | 0.19 | 0.22 | 0.69 | 0.84 (0.73-0.96) | 1.23 × 10^-2^ | 0.403 |
| rs7456261 | *ATP2B2* | 7 | 10656822 | 1.00 | A/G | 0.16 | 0.12 | 0.80 | 1.34 (1.14-1.57) | 3.52 × 10^-4^ | 0.107 |
| rs76982728 | *PDE1C* | 7 | 32316812 | 0.96 | T/C | 0.33 | 0.30 | 0.54 | 1.16 (1.02-1.31) | 2.16 × 10^-2^ | 0.452 |
| rs7778364 | *PDE1C* | 7 | 32111001 | 1.00 | C/T | 0.19 | 0.16 | 0.68 | 1.25 (1.08-1.44) | 3.18 × 10^-3^ | 0.227 |
| rs7778749 | *GRIN2A* | 7 | 9928865 | 1.00 | C/A | 0.19 | 0.16 | 0.37 | 1.18 (1.03-1.37) | 2.19 × 10^-2^ | 0.436 |
| rs7779058 | *ATP2B2* | 7 | 10576197 | 0.95 | A/G | 0.08 | 0.06 | 0.21 | 1.40 (1.13-1.75) | 2.47 × 10^-3^ | 0.200 |
| rs9639635 | *PDE1C* | 7 | 32463994 | 0.96 | A/G | 0.11 | 0.08 | 0.55 | 1.40 (1.15-1.70) | 9.79 × 10^-4^ | 0.149 |
| rs979356 | *PDE1C* | 7 | 32424383 | 0.97 | A/T | 0.24 | 0.22 | 1.00 | 1.15 (1.00-1.31) | 4.33 × 10^-2^ | 0.510 |
| rs10102186 | *ADRA1A* | 8 | 26624650 | 1.00 | A/G | 0.05 | 0.07 | 0.27 | 0.73 (0.58-0.93) | 1.15 × 10^-2^ | 0.389 |
| rs4732902 | *RYR3* | 8 | 33967373 | 1.00 | G/A | 0.29 | 0.26 | 0.06 | 1.14 (1.01-1.29) | 4.04 × 10^-2^ | 0.511 |
| rs12775181 | *HTR7* | 10 | 92564456 | 1.00 | C/G | 0.11 | 0.09 | 0.61 | 1.28 (1.06-1.55) | 1.03 × 10^-2^ | 0.358 |
| rs141748886 | *RYR1* | 10 | 39067516 | 0.96 | G/T | 0.27 | 0.25 | 0.11 | 1.15 (1.00-1.31) | 4.46 × 10^-2^ | 0.521 |
| rs12579387 | *NOS1* | 12 | 117763450 | 0.99 | A/G | 0.22 | 0.24 | 1.00 | 0.85 (0.74-0.97) | 1.75 × 10^-2^ | 0.433 |
| rs816347 | *GRIN2A* | 12 | 10059778 | 0.99 | A/G | 0.08 | 0.06 | 0.13 | 1.32 (1.06-1.64) | 1.37 × 10^-2^ | 0.426 |
| rs816358 | *RYR1* | 12 | 38924813 | 1.00 | T/G | 0.06 | 0.05 | 0.38 | 1.28 (1.01-1.61) | 4.28 × 10^-2^ | 0.510 |
| rs877995 | *P2RX6* | 12 | 21377952 | 0.97 | A/G | 0.11 | 0.13 | 1.00 | 0.83 (0.70-0.99) | 3.54 × 10^-2^ | 0.495 |
| rs9658490 | *NOS1* | 12 | 31750680 | 0.99 | G/C | 0.09 | 0.07 | 0.69 | 1.27 (1.04-1.55) | 1.98 × 10^-2^ | 0.446 |
| rs9658525 | *NOS1* | 12 | 117221948 | 0.98 | T/C | 0.07 | 0.06 | 0.79 | 1.37 (1.10-1.72) | 5.94 × 10^-3^ | 0.301 |
| rs11635223 | *RYR3* | 15 | 34014752 | 1.00 | T/C | 0.20 | 0.17 | 0.85 | 1.17 (1.01-1.35) | 3.29 × 10^-2^ | 0.494 |
| rs12324083 | *RYR3* | 15 | 33653510 | 0.97 | C/A | 0.11 | 0.09 | 0.59 | 1.30 (1.07-1.57) | 7.81 × 10^-3^ | 0.316 |
| rs12440440 | *RYR3* | 15 | 34041895 | 1.00 | A/G | 0.31 | 0.28 | 0.46 | 1.13 (1.00-1.28) | 4.63 × 10^-2^ | 0.525 |
| rs12913815 | *RYR3* | 15 | 34001141 | 0.98 | T/G | 0.11 | 0.08 | 0.69 | 1.50 (1.23-1.82) | 6.27 × 10^-5^ | 0.038 |
| rs146559815 | *ADCY2* | 15 | 7538101 | 0.99 | T/A | 0.17 | 0.14 | 0.82 | 1.26 (1.08-1.47) | 3.72 × 10^-3^ | 0.238 |
| rs1565937 | *RYR3* | 15 | 33791242 | 1.00 | C/T | 0.28 | 0.25 | 0.31 | 1.14 (1.00-1.29) | 4.63 × 10^-2^ | 0.521 |
| rs16953863 | *GRIN2A* | 15 | 10002808 | 1.00 | G/A | 0.23 | 0.26 | 0.40 | 0.83 (0.73-0.94) | 4.42 × 10^-3^ | 0.269 |
| rs16971757 | *HTR4* | 15 | 147998118 | 1.00 | A/G | 0.25 | 0.30 | 0.47 | 0.79 (0.70-0.89) | 2.13 × 10^-4^ | 0.086 |
| rs3829480 | *ADRA1B* | 15 | 159381525 | 1.00 | C/T | 0.44 | 0.46 | 0.44 | 0.89 (0.79-0.99) | 3.93 × 10^-2^ | 0.503 |
| rs4779631 | *RYR3* | 15 | 33898072 | 1.00 | A/G | 0.33 | 0.31 | 0.85 | 1.14 (1.01-1.29) | 3.24 × 10^-2^ | 0.493 |
| rs4780150 | *ADRA1D* | 15 | 4200273 | 1.00 | T/C | 0.11 | 0.13 | 0.80 | 0.83 (0.69-0.99) | 3.40 × 10^-2^ | 0.480 |
| rs57050063 | *RYR1* | 15 | 39072161 | 0.99 | A/G | 0.18 | 0.15 | 0.91 | 1.28 (1.10-1.49) | 1.42 × 10^-3^ | 0.157 |
| rs684678 | *RYR3* | 15 | 33781322 | 0.99 | A/G | 0.24 | 0.21 | 0.62 | 1.17 (1.03-1.34) | 1.80 × 10^-2^ | 0.428 |
| rs7167430 | *PDE1C* | 15 | 31934217 | 1.00 | C/T | 0.2 | 0.24 | 0.37 | 0.79 (0.69-0.90) | 5.77 × 10^-4^ | 0.100 |
| rs74005913 | *GRIN2A* | 15 | 10134585 | 0.99 | G/A | 0.08 | 0.06 | 0.60 | 1.36 (1.09-1.71) | 7.13 × 10^-3^ | 0.321 |
| rs8025855 | *NOS1* | 15 | 117697307 | 1.00 | C/T | 0.22 | 0.19 | 0.79 | 1.21 (1.06-1.39) | 6.65 × 10^-3^ | 0.311 |
| rs8028974 | *NOS1* | 15 | 117663249 | 1.00 | G/A | 0.17 | 0.14 | 0.58 | 1.21 (1.04-1.42) | 1.58 × 10^-2^ | 0.416 |
| rs11642171 | *GRIN2A* | 16 | 10271163 | 0.96 | A/C | 0.43 | 0.47 | 0.96 | 0.85 (0.76-0.95) | 5.10 × 10^-3^ | 0.295 |
| rs1237957 | *GRIN2A* | 16 | 10096869 | 0.98 | C/G | 0.34 | 0.36 | 0.77 | 0.88 (0.79-0.99) | 3.78 × 10^-2^ | 0.499 |
| rs17208905 | *ERBB4* | 16 | 212239074 | 0.96 | G/A | 0.10 | 0.08 | 0.17 | 1.26 (1.03-1.53) | 2.43 × 10^-2^ | 0.433 |
| rs1875205 | *P2RX6* | 16 | 21372159 | 1.00 | C/A | 0.34 | 0.37 | 0.56 | 0.87 (0.78-0.98) | 2.21 × 10^-2^ | 0.434 |
| rs2352749 | *ADCY2* | 16 | 7466175 | 1.00 | A/T | 0.34 | 0.37 | 0.52 | 0.87 (0.78-0.98) | 2.39 × 10^-2^ | 0.454 |
| rs2650432 | *GRIN2A* | 16 | 10083882 | 1.00 | G/A | 0.31 | 0.36 | 0.55 | 0.81 (0.72-0.91) | 4.85 × 10^-4^ | 0.098 |
| rs28789085 | *GRIN2A* | 16 | 10094137 | 0.99 | T/C | 0.38 | 0.35 | 0.12 | 1.15 (1.02-1.28) | 1.99 × 10^-2^ | 0.440 |
| rs3105692 | *ERBB4* | 16 | 212845416 | 0.98 | G/C | 0.4 | 0.44 | 0.24 | 0.84 (0.75-0.94) | 2.22 × 10^-3^ | 0.207 |
| rs3859123 | *ATP2B2* | 16 | 10632678 | 1.00 | G/A | 0.43 | 0.4 | 0.28 | 1.15 (1.03-1.28) | 1.45 × 10^-2^ | 0.421 |
| rs74632430 | *RYR1* | 16 | 39052107 | 0.97 | T/C | 0.38 | 0.35 | 0.24 | 1.15 (1.02-1.29) | 2.18 × 10^-2^ | 0.442 |
| rs78174306 | *ADCY2* | 16 | 7509617 | 0.96 | C/T | 0.12 | 0.1 | 0.74 | 1.24 (1.03-1.49) | 2.17 × 10^-2^ | 0.446 |
| rs884917 | *PDE1C* | 16 | 31790293 | 0.99 | G/A | 0.39 | 0.42 | 0.78 | 0.86 (0.77-0.96) | 7.90 × 10^-3^ | 0.310 |
| rs11078474 | *P2RX1* | 17 | 3815030 | 0.96 | T/C | 0.19 | 0.17 | 0.76 | 1.17 (1.01-1.35) | 3.71 × 10^-2^ | 0.495 |
| rs10408694 | *RYR1* | 19 | 39075391 | 1.00 | C/T | 0.25 | 0.22 | 0.87 | 1.17 (1.02-1.33) | 2.40 × 10^-2^ | 0.441 |
| rs10409638 | *RYR1* | 19 | 38975295 | 1.00 | G/A | 0.21 | 0.19 | 0.66 | 1.15 (1.00-1.32) | 4.66 × 10^-2^ | 0.520 |
| rs1468570 | *ERBB4* | 19 | 213215935 | 0.95 | A/G | 0.09 | 0.06 | 0.48 | 1.48 (1.19-1.83) | 4.13 × 10^-4^ | 0.100 |
| rs2116869 | *RYR1* | 19 | 32456845 | 0.99 | G/A | 0.12 | 0.15 | 1.00 | 0.83 (0.70-0.98) | 2.43 × 10^-2^ | 0.428 |
| rs307954 | *CHRM2* | 19 | 136669439 | 0.96 | A/T | 0.29 | 0.32 | 0.95 | 0.87 (0.77-0.99) | 3.39 × 10^-2^ | 0.484 |
| rs59812056 | *ERBB4* | 19 | 212573894 | 0.98 | G/A | 0.17 | 0.13 | 0.47 | 1.29 (1.10-1.52) | 1.57 × 10^-3^ | 0.159 |
| rs75797287 | *CAMK2B* | 19 | 44312823 | 0.95 | T/C | 0.19 | 0.15 | 0.58 | 1.26 (1.09-1.47) | 2.38 × 10^-3^ | 0.207 |
| rs919781 | *NOS1* | 19 | 117670297 | 1.00 | T/C | 0.13 | 0.11 | 0.18 | 1.20 (1.01-1.43) | 3.56 × 10^-2^ | 0.492 |
| rs59616334 | *PDE1C* | 20 | 32329945 | 0.98 | T/C | 0.32 | 0.29 | 0.25 | 1.14 (1.01-1.28) | 4.17 × 10^-2^ | 0.517 |
| rs2073597 | *P2RX6* | 22 | 32447343 | 0.97 | T/G | 0.25 | 0.22 | 0.07 | 1.18 (1.03-1.35) | 1.87 × 10^-2^ | 0.436 |
| rs9625356 | *NOS1* | 22 | 117659752 | 0.97 | T/C | 0.37 | 0.34 | 0.85 | 1.15 (1.02-1.30) | 2.10 × 10^-2^ | 0.447 |

OR, odds ratio; CI, confidence interval; MAF, minor allele frequency.

^a^ Reference/effect allele.

^b^ HWE: Hardy Weinberg Equilibrium in controls.

^c^ Adjusted for age and sex in logistic regression model.
