# Supplementary material for: Genetic variants in the calcium signaling pathway participate in the pathogenesis of colorectal cancer through the tumor microenvironment
Source: Front Oncol. 2023 Feb 7;13:992326. doi: 10.3389/fonc.2023.992326 (PMC9941622; doi:10.3389/fonc.2023.992326)
Supplement: Supplementary file 9 [file Table_3.docx]

**Supplementary Table 3. Functional annotation for rs12538364**

| SNPs | Rank^a^ | DNAse^b^ | Motifs changed |
| --- | --- | --- | --- |
| rs12538364 | 4 | OVARY | 4 altered motifs |
| rs12913815 | 5 |  | 3 altered motifs |

^a^ Based on RegulomeDB

^b^ Based on HaploReg v4.1
