# Supplementary material for: Genetic variants in the calcium signaling pathway participate in the pathogenesis of colorectal cancer through the tumor microenvironment
Source: Front Oncol. 2023 Feb 7;13:992326. doi: 10.3389/fonc.2023.992326 (PMC9941622; doi:10.3389/fonc.2023.992326)
Supplement: Supplementary file 10 [file Table_4.docx]

**Supplementary Table 4. Stratified analysis of clinicopathologic characteristics for the association between *PDE1C* rs12538364 and colorectal cancer risk**

| Variables | Category |  | Cases |  | Adjusted OR (95%CI)^a^ | *P*^a^ | *P*^b^ |
| --- | --- | --- | --- | --- | --- | --- | --- |
|  |  | CC | CT | TT |  |  |  |
| Tumor grade | Well + moderate | 750 (77.3) | 209 (21.5) | 11 (1.2) | 1.57 (1.29-1.91) | 8.70 × 10^-6^ | 0.879 |
|  | poor | 136 (77.3) | 38 (21.6) | 2 (1.1) | 1.62 (1.14-2.31) | 7.70 × 10^-3^ |  |
| Tumor site | colon | 447 (76.5) | 132 (22.6) | 5 (1.0) | 1.62 (1.29-2.03) | 3.64 × 10^-5^ | 0.729 |
|  | Rectum | 439 (78.1) | 115 (20.5) | 8 (1.4) | 1.53 (1.21-1.92) | 3.12 × 10^-4^ |  |
| Tumor stage | A+B | 389 (76.4) | 112 (22.0) | 8 (1.6) | 1.65 (1.31-2.08) | 2.83 × 10^-5^ | 0.619 |
|  | C+D | 497 (78.0) | 135 (21.2) | 5 (21.2) | 1.52 (1.21-1.90) | 3.04 × 10^-4^ |  |

OR odds ratio, *CI* confidence interval

^a^ Adjusted for age and sex in the logistic regression model

^b^ *P* value for the heterogeneity
